# An Updated Review of Lysophosphatidylcholine Metabolism in Human Diseases

**DOI:** 10.3390/ijms20051149

**Published:** 2019-03-06

**Authors:** Shi-Hui Law, Mei-Lin Chan, Gopal K. Marathe, Farzana Parveen, Chu-Huang Chen, Liang-Yin Ke

**Affiliations:** 1 Department of Medical Laboratory Science and Biotechnology, College of Health Sciences, Kaohsiung Medical University, Kaohsiung 80708, Taiwan; shlaw_0909@hotmail.com (S.-H.L.); farzanaparveen@zoho.com (F.P.); 2 Center for Lipid Biosciences, Kaohsiung Medical University Hospital, Kaohsiung 80708, Taiwan; mlchan127@gmail.com (M.-L.C.); cchen@texasheart.org (C.-H.C.); 3 Division of Thoracic Surgery, Department of Surgery, MacKay Memorial Hospital, MacKay Medical College, Taipei 10449, Taiwan; 4 Department of Studies in Biochemistry, Manasagangothri, University of Mysore, Mysore 570006, India; marathe1962@gmail.com; 5 Lipid Science and Aging Research Center, Kaohsiung Medical University, Kaohsiung 80708, Taiwan; 6 Graduate Institute of Medicine, College of Medicine, Kaohsiung Medical University, Kaohsiung 80708, Taiwan; 7 Vascular and Medicinal Research, Texas Heart Institute, Houston, TX 77030, USA

**Keywords:** lysophosphatidylcholine, lipoprotein-associated phospholipase A_2_, lysophosphatidylcholine acyltransferase, lysophospholipase A_1_, autotaxin, G protein–coupled receptor G2A

## Abstract

Lysophosphatidylcholine (LPC) is increasingly recognized as a key marker/factor positively associated with cardiovascular and neurodegenerative diseases. However, findings from recent clinical lipidomic studies of LPC have been controversial. A key issue is the complexity of the enzymatic cascade involved in LPC metabolism. Here, we address the coordination of these enzymes and the derangement that may disrupt LPC homeostasis, leading to metabolic disorders. LPC is mainly derived from the turnover of phosphatidylcholine (PC) in the circulation by phospholipase A_2_ (PLA_2_). In the presence of Acyl-CoA, lysophosphatidylcholine acyltransferase (LPCAT) converts LPC to PC, which rapidly gets recycled by the Lands cycle. However, overexpression or enhanced activity of PLA_2_ increases the LPC content in modified low-density lipoprotein (LDL) and oxidized LDL, which play significant roles in the development of atherosclerotic plaques and endothelial dysfunction. The intracellular enzyme LPCAT cannot directly remove LPC from circulation. Hydrolysis of LPC by autotaxin, an enzyme with lysophospholipase D activity, generates lysophosphatidic acid, which is highly associated with cancers. Although enzymes with lysophospholipase A_1_ activity could theoretically degrade LPC into harmless metabolites, they have not been found in the circulation. In conclusion, understanding enzyme kinetics and LPC metabolism may help identify novel therapeutic targets in LPC-associated diseases.

## 1. Introduction

### 1.1. General Features of Lysophosphatidylcholine

Lysophosphatidylcholine (LPC), also called lysolecithins, is a class of lipid biomolecule derived by the cleaving of phosphatidylcholine (PC) via the action of phospholipase A_2_ (PLA_2_) [1,2] and/or by the transfer of fatty acids to free cholesterol via lecithin-cholesterol acyltransferase (LCAT) [3]. In healthy individuals, the plasma level of LPC ranges from 125 to 143 nmole/mL, but its level increases in cardiovascular diseases, diabetes, ovarian cancer, and renal failure [4,5,6]. PC, also called lecithins (*lécithine*), was originally derived from the Greek word “*lekithos*” (λεκιθος, egg yolk), and in 1847, Theodore Nicolas Gobley [7] published a description of their chemical structure. PC is a major component of biological membranes found in animal and plant cells [8]. Although LPCs can be produced in the circulation when PLA_2_ cleaves PCs, they can be converted back to PCs by the enzyme lysophosphatidylcholine acyltransferase (LPCAT) in the presence of Acyl-CoA. These two pathways are part of the Lands cycle [9], which is one of the body’s mechanisms for the cyclical synthesis and degradation of PC. The details of these processes are described in this review. LPCATs are intracellular enzymes found in body tissues such as lung [10], liver [11], and adipose tissue [12], but these intracellular enzymes are unlikely to interact directly with extracellular circulating LPC, which is positively associated with diseases [4,5,6].

### 1.2. Effects of Lysophosphatidylcholines

In the liver, LPCs upregulate genes involved in cholesterol biosynthesis and downregulate genes involved in hepatic fatty acid oxidation [13]. Higher concentrations of LPCs disrupt mitochondrial integrity and enhance cytochrome C release in hepatocytes (Table 1). In the vascular system, LPC induces prolonged endothelial activation and atherogenesis [14,15]. It modulates inflammatory chemokine expression from endothelial cells [16,17,18,19], impairs arterial relaxation [20], increases oxidative stress [21,22], and inhibits endothelial cell migration and proliferation [23,24]. Rich in oxidized low-density lipoprotein (OxLDL), LPCs have been identified as a group of proinflammatory lipids that are critically involved in the pathogenesis of atherosclerosis [25] and other inflammatory diseases such as multiple sclerosis [26,27]. Overproduction of LPC can result from the overexpression or enhanced activity of enzymes such as lipoprotein-associated phospholipase A_2_ (Lp-PLA_2_) in circulation [28,29]. In contrast, effectively clearing LPC, through either Lands cycle remodeling inside cells or the direct degradation of circulating LPC, is essential for maintaining LPC levels.

### 1.3. Lysophosphatidylcholine Signaling through Receptors

LPC activates multiple signaling pathways that are involved in oxidative stress and inflammatory responses. The signaling cascade is triggered through G protein–coupled receptor G2A [30,31,32,33] and Toll-like receptors [34]. However, whether G2A is a canonical receptor for LPC is debated [35]. It is notable that LPC is not an agonist of the platelet-activating factor receptor, and the LPC used in previous studies may have been contaminated with PAF-like lipids [36]. Exogenous LPC induces pro-inflammatory effects such as upregulated gene expression for smooth muscle/fibroblast-directed growth factors and adhesion molecules in endothelial cells [37,38], increased release of interleukin-1β (IL-1β), IL-6, and tumor necrosis factor-α (TNF-α) from adipocytes [39,40], enhanced secretion of interferon-γ from peripheral blood mononuclear leucocytes [16], and increased activation of B cells [16] and macrophages [30,34,41]. In addition, LPC enhances the Foxp3 expression and suppressive function of naturally occurring regulatory T cells (nTregs) [42]; these actions are believed to be mediated through G2A signaling [42]. nTregs are responsible for preventing immune responses through various means, including the production of anti-inflammatory cytokines [43]. In 2014, researchers reported that LPC promotes and stabilizes macrophage polarization strongly toward the M1 phenotype, and that blocking the G2A receptor reduces the impact of LPC on macrophage polarization [44]. In the central nervous system, LPC induces demyelination in white matter of the spinal cord by activating G protein-coupled receptor 17 (*Gpr17*) signaling. Highly expressed in brain tissue, GPR17 reduces the intracellular cAMP level and induces pro-apoptotic gene XIAP-associated factor 1 (Xaf1) expression, which in turn inhibits oligodendrocyte survival and precursor cell differentiation [45]. In another study, LPC impaired the barrier function of the endothelium in the brain microvasculature and induced inflammation through G protein-coupled receptor4 (Gpr4) [46]. LPC mediates pericyte loss, vascular barrier disruption, demyelination, and motor function defects; all of these effects can be decreased by iloprost, an analog of prostacyclin [47]. The mechanism for demyelination involves the integration of LPC into the cell membrane, which induces permeability and necrotic cell death [48].

### 1.4. Recent Clinical Findings of Lysophosphatidylcholines

As described above, LPC may alter the physiology of the vascular endothelium, pericytes, and neuron cells in vitro and in vivo, indicating it may be a compelling risk factor and may be associated with the pathogenesis and prognosis of cardiovascular diseases. However, findings from recent clinical lipidomics studies have been controversial and somewhat confusing. For example, plasma LPCs showed an inverse relationship with cardiovascular diseases [51,52,53]. In other studies, the LPC:PC ratio decreased either in plasma or cerebrospinal fluid from patients with Alzheimer’s disease [54,55,56]. To help clarify the controversy and delineate the role of LPC in these diseases, we have provided a timely and important updated review on LPC homeostasis. In this article, we discuss whether excess LPC has a cause-effect association with human diseases. Specifically, we review LPC production in circulation and its transport and reconversion to PC within cells.

## 2. Lysophosphatidylcholine and Human Diseases

### 2.1. Lysophosphatidylcholine and Cardiovascular Diseases

Cardiovascular diseases are a class of diseases that include atherosclerosis, diabetes, metabolic syndrome, myocardial infarction, and angina. According to the World Health Organization, cardiovascular diseases account for 17.7 million deaths (31% of all global deaths) each year and are now the leading cause of death in the world [57]. Atherosclerosis is a pathological process that involves plaque build-up in the walls of the arteries [58]. LPC levels in the circulation are associated with the development of atherosclerotic plaques and endothelial cell dysfunction [58,59,60,61]. Some LPC species can be diagnostic markers for myocardial infarction [62]. LPC content is increased in circulating modified low-density lipoprotein (LDL) [63], enzymatically degraded LDL [64], and oxidized LDL [64,65]. In addition, LPC promotes fatty acid-induced insulin resistance [66] and inhibits endothelial progenitor cell revitalization [67]. LPC and LDL levels are increased in the plasma of patients with familial hyperlipidemia and diabetes [65,68,69] (Table 2), and treatment with simvastatin reduces Lp-PLA_2_ and LPC content [68]. Interestingly, several recent lipidomic profiling studies showed a negative correlation between LDL levels and the occurrence of cardiovascular diseases [51,52,53]. In addition, diabetes is an important risk factor for cardiovascular diseases; however, conflicting results have been reported on the correlation of LPC and diseases [4,70,71,72,73].

### 2.2. Lysophosphatidylcholine and Brain Diseases

Oligodendrocytes are myelin-producing cells that provide metabolic support for neurons and prevent neurodegeneration [78]. Myelination defects are seen in many brain diseases such as multiple sclerosis, stroke, schizophrenia, and Alzheimer’s disease [47,79]. LPC mediates pericyte loss, vascular barrier disruption, demyelination, and motor function defects [45,46,47]. In addition, LPC enhances the neurotoxicity of amyloid β_1–42_ peptide oligomer formation and neurotoxic protein aggregation, indicating that inhibiting LPC generation may be important in treating neurodegenerative diseases [80,81]. In one study, LPC levels were significantly increased in patients with repetitive mild traumatic brain injury [82]. However, other studies have shown that plasma levels of LPC were decreased in patients with Alzheimer’s disease [54,55] and that the LPC-to-PC ratio was also decreased either in plasma or in cerebrospinal fluid from patients with Alzheimer’s disease [56,77].

### 2.3. Brief Summary of Lysophosphatidylcholine in Human Disease

Through Notch1 and/or ERK1/2 signaling, LPC induces monocyte chemoattractant protein-1 and inflammatory cytokine expression and damages endothelial cells [18,19,50]. In addition, LPC activates monocytes and polarizes macrophage activation toward the M1 phenotype [30,34,41,44], leading to the development of atherosclerosis and cardiovascular diseases. In the brain, LPC promotes oligodendrocyte demyelination and pericyte loss, and impairs barrier function of the endothelium [45,46,47,48], leading to neurodegenerative diseases. LPC levels are determined by different mechanisms, including LPC production, clearance, and degradation. Overproduction of LPC and/or increasing LPC levels in LDL particles, or in tissue, are positively correlated with disease development.

## 3. Mechanisms for Increased Circulating Lysophosphatidylcholine Levels

### 3.1. Increased Degradation of Phosphatidylcholine by Lipoprotein-Associated Phospholipase A_2_

**Figure 1:**
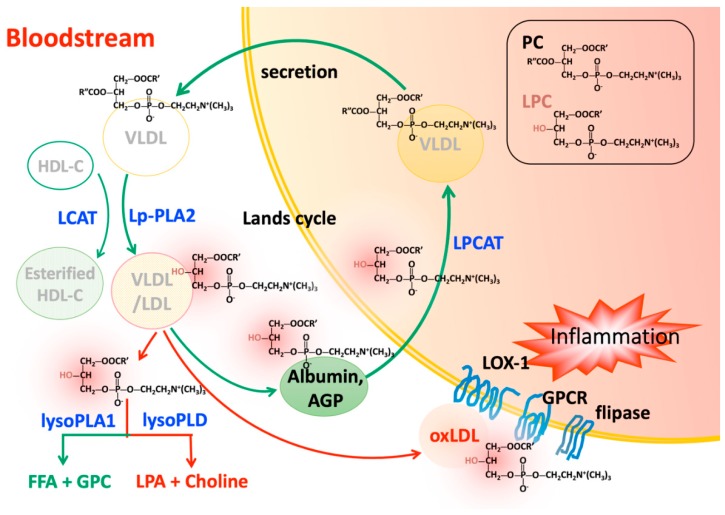
Phosphatidylcholines (PCs) are synthesized in the liver and secreted as components of very low density lipoprotein (VLDL). PCs are degraded via hydrolysis by Lp-PLA2 or conversion by LCAT. The metabolites, LPCs, can be transported back to the liver by albumin or AGP and then cleared by LPCAT in the presence of acyl-CoA. The actions of these two distinct enzymes form a cycle of PC degradation and regeneration called the Lands cycle. Excess circulating LPCs may be released or carried on OxLDL to exert harmful effects on various cells though LOX-1, lipid flippase, and G protein-coupled receptor signaling. LPCs can also undergo further hydrolysis by lysoPLD such as autotaxin to become LPA, another important inflammatory mediator. Green arrows indicate occurrence under normal physiologic conditions. Red arrows indicate the promotion of inflammation.

PC synthesized in the liver is the most abundant lipid component (up to 70% mole ratio) of plasma very low density lipoprotein (VLDL) and also makes up close to 40% of nascent high-density lipoprotein (HDL) [83,84]. In the liver, PC is involved in VLDL secretion [85,86,87] and HDL metabolism [88,89]. After being secreted into the blood stream, PC on lipoprotein particles is degraded at the Sn-2 position of an oxidized fatty acid by the hydrolysis of Lp-PLA_2_ (Figure 1). LPC is then produced under a variety of physiological and pathological conditions. In atherosclerotic plaque, macrophages produce Lp-PLA_2_, which is then secreted into the circulatory system [90]. ApoCIII, OxLDL, serum amyloid A, and leukocyte activation are associated with the regulation and activation of Lp-PLA_2_ expression [91,92,93]. In contrast, nitro-oleic acid downregulates Lp-PLA_2_ expression [94]. Extensive clinical evidence indicates that the quantity and activity of Lp-PLA_2_ are positively correlated with cardiovascular events [27,95,96,97]. Quantifying plasma Lp-PLA_2_ is useful for identifying plaque instability, acute coronary syndrome, and other cardiovascular diseases [98,99,100]. Moreover, Lp-PLA_2_ is a predictor for incident ischemic stroke severity, early neurological deterioration in patients with acute ischemic stroke, and delayed cerebral ischemia in patients with aneurysmal subarachnoid hemorrhage [101,102,103]. However, inhibiting Lp-PLA_2_ by darapladib, a synthetic specific small molecular weight inhibitor of platelet-activating factor-acetylhydrolase (PAF-AH), did not yield promising results in clinical trials [104,105]. Although Lp-PLA_2_ has the anti-inflammatory function of degrading PAF [106,107] and thus reduces platelet activation, it also has the proinflammatory properties of increasing LPC and oxidized non-esterified fatty acids levels, which may be associated with the development of atherosclerosis [16,108]. Another potential future strategy for inhibiting Lp-PLA_2_ may involve the use of combined RNA interference (RNAi), which ameliorated atherosclerosis in apolipoprotein E-deficient mice [109].

### 3.2. Increased Degradation of Phosphatidylcholine by Lecithin-Cholesterol Acyltransferase

LCAT is secreted from the liver and catalyzes the transfer of the fatty acids at position sn-2 of PC to free cholesterol in plasma, which results in the formation of cholesterol esters and LPC on the surface of HDL and LDL [110,111] (Figure 1). In the brain, LCAT is synthesized by primary astrocytes and is activated by apolipoprotein E (apoE) secreted by glial cells [112]. The effect of LCAT on human atherogenesis is controversial [113,114]. Genetic deficiency of LCAT leads to the accumulation of nascent pre-β HDL in the circulation and development of atherosclerosis and acute coronary syndrome [114,115,116,117,118,119,120,121]. In golden Syrian hamsters, the loss of LCAT activity led to dyslipidemia and atherosclerosis [122]. In addition, proteomic studies in LCAT deficiency showed that plasma levels of HDL-C, apoAI, and apoAI were decreased, leading to corneal opacity, hemolytic anemia, and renal disease [123]. In contrast, overexpression of LCAT is associated with the formation of large apoE-rich HDL and liver cholesterol [124,125]. Higher LCAT activity is correlated with insulin resistance and nonalcoholic fatty liver disease [126]. The LDL-particle size can be reduced and positively associated with atherosclerotic cardiovascular disease [127]. However, in some cases, increased LCAT levels are associated with a reduced coronary atheroma burden [128]. Interestingly, LCAT levels did not differ in LCAT-transgenic mice and wild-type mice [129]. The detailed mechanisms are unclear.

### 3.3. Hypoxia Condition Regulates Glycolysis and Lysophosphatidylcholine Overproduction

Hypoxia induces a reprogramming of cell respiration and carbohydrate metabolism [130]. Under hypoxic conditions, hypoxia-inducible factor (HIF)-1 alters the expression profile of glycolytic molecules and regulates glycolysis [131]. In addition, HIF-1 in the liver activates sterol regulatory element binding protein-1 and stearoyl-coenzyme A desaturase, which are key regulatory genes in the biosynthesis of triglycerides and phospholipids [132]. Trzepizur et al. examined serum lipid levels in 2018 fasting patients and found that nocturnal intermittent hypoxia and obstructive sleep apnea severity were associated with higher triglyceride and lower HDL-C levels [133]. In cardiovascular diseases, intermittent hypoxia leads to cardiomyocyte apoptosis and inflammation through protein *O*-GlcNAc glycosylation and phosphorylation of the p38 mitogen-activated protein kinase [134]. Some lipidomic studies showed that hypoxia stimulation caused a prominent increase in the amount of LPC [135]. In addition, findings from a lipid consumption study also suggested that LPC provides a more accessible nutrient source for cell proliferation under hypoxia [136]. In AbPP^Swe^/PSI^dE9^ mice, chronic intermittent hypoxia triggered earlier learning memory impairment. In a symptomatic N5 TgCRND8 mouse model of Alzheimer’s disease, cytosolic phospholipase A2α activity progressively increased; overall LPC levels progressively rose. The authors concluded that disruptions in Lands cycle metabolism were linked to the onset of symptoms and a progressive behavioral decline in mice with pre-existing Aβ pathology [137]. However, the detailed effects of hypoxia on lipid metabolism in humans are not well understood.

## 4. Transportation of Lysophosphatidylcholine in the Circulatory System

Plasma LPC is rapidly cleared from circulation by transporters such as albumin and alpha-1 acid glycoprotein (AGP) to the liver for the synthesis of PC [138], or it accumulates in the brain for the production of acetylcholine [139] (Figure 1). Although overproduction of LPC can occur through different mechanisms as mentioned above, it can also accumulate in oxidized LDL and is associated with vascular inflammation [140,141].

### 4.1. Albumin

About 80% of LPC is bound to albumin [142,143]. Hypoalbuminemia due to proteinuria results in a decrease in albumin-LPC binding and an increase in LPC levels in VLDL, intermediate-density lipoprotein, and LDL [144]. Increased levels of LPC in LDL of hyperlipidemic patients were associated with nephrotic syndrome [145]. In another study, albumin was found to be protective in LPC-induced attenuation of vasodilation [146]. Moreover, albumin counteracted LPC-induced renal vasoconstriction [147], suggesting that albumin is a potent buffer for the effects of LPC.

### 4.2. Alpha-1 Acid Glycoprotein

The concentration of AGP in the plasma increases under inflammatory conditions. Similar to the function of antitrypsin, AGP exerts anti-inflammatory effects by inhibiting platelet aggregation, preventing superoxide production, and attenuating TNF-α effects on cells [148]. In addition, AGP maintains capillary permeability and prevents apoptosis in ischemia/reperfusion injury. With a higher binding affinity to LPC, AGP complements albumin as a lysophospholipid-scavenging protein, especially in inflammatory conditions in which albumin-sequestering capacity is weakened [138].

### 4.3. Transmembrane Transporter Protein

P4-ATPases are lipid flippases expressed on the eukaryotic plasma membrane. They function in translocating phospholipids from the exoplasm to the cytosolic area against a concentration gradient via ATP hydrolysis [149,150]. In a reverse process, LPC can be exported by the action of ATP-binding cassette transporter A7 (ABCA7) in the presence of apolipoprotein AI (apoAI) and apoE [151,152]. Mutations of ABCA7 are associated with neurodegenerative diseases [153]. The possible mechanism may involve a scenario in which excess LPC synergistically enhances Aβ1-42-induced neuronal apoptosis [154,155].

### 4.4. Oxidized Low Density Liopoprotein

Human lipoproteins, including VLDL, LDL, and HDL, contain LPC. However, the levels of LPC in these lipoproteins are debated [65,156,157]. Using electrospray ionization and matrix-assisted laser desorption/ionization mass spectrometry, Stübiger et al. reported that LPC concentration was increased in patients with familial hypercholesterolemia or familial combined hyperlipidemia [65]. Specifically, the amount of bioactive lipid LPC is increased up to 5 times in circulating modified LDL or OxLDL [63,65,158]. Because of the atherogenicity of LPC, OxLDL modulates dendritic cell phenotypic and functional maturation [159,160], triggers adipocyte activation and plasminogen activator inhibitor-1 secretion [40], leads to endothelial damage by inhibiting Ca^2+^ influx and NO synthesis [161], and promotes human artery smooth muscle cells proliferation and migration [162]. These pathophysiological implications make LPC a promising target for biomarker and treatment of atherosclerosis and cardiovascular disorders [162,163].

## 5. Lysophosphatidylcholine Turnover

### 5.1. Lysophosphatidylcholine Clearance by Acyltransferases in Various Tissues

Under normal physiological conditions, LPC is cleared by enzymes such as acyl-CoA:LPCAT [164], located in the endoplasmic reticulum within alveolar type II cells in the lung [10], in lipid droplets [12], in red blood cells [164], in hepatocytes [11,165], and in other cell types (Figure 1). At least four LPCAT subtypes have been identified [166]. Among them, overexpression of the *LPCAT1* gene may contribute to the progression and metastasis of human cancers, such as hepatocellular carcinoma [167], oral squamous cell carcinoma [168], breast cancer [169], prostate cancer [170], and colorectal cancer [171]. LPCAT2 supports lipid droplet production, and its overexpression inhibits the function of chemotherapeutic agents for colorectal cancer [172]. Expression of the *LPCAT2* gene is upregulated in breast and cervical cancers [173]. *LPCAT3* is regulated by peroxisome proliferator-activated receptor δ. Transient liver-specific knockdown of *LPCAT3* in mice attenuated the fatty acid metabolic pathway [11,165]. In another study, *LPCAT3* knockdown resulted in LPC accumulation in the liver but promoted VLDL secretion and microsomal triglyceride transfer protein expression [174]. In addition, *LPCAT3* deficiency reduced lipid adsorption in small intestine [175]. LPCAT4 is also called acyl-CoA:lysophosphatidylethanolamine acyltransferase 2 and is primarily expressed in the brain [176]. In colorectal cancer, LPCAT4 levels are elevated [177]. Tumor necrosis factor-α and transforming growth factor-β1 induced the expression of LPCAT2 and LPCAT4 [178,179].

### 5.2. Degradation of Lysophosphatidylcholine by Lysophospholipases in the Circulation

**Figure 2:**
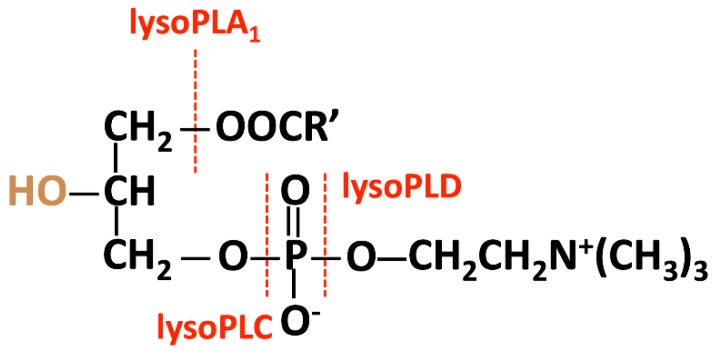
The hydrolysis of LPC is catalyzed by lysophospholipases A1, C, or D, according to the cleavage site.

The hydrolysis of LPC can be catalyzed by lysophospholipases A_1_, C, or D, according to the cleavage site (Figure 2). In neutrophils in humans, phospholipase B-like 1 exhibits weak lysophospholipase A_1_ activity [180]. Autotaxin has lysophospholipase D activity; the product resulting from the action of autotaxin—lysophosphatidic acid (LPA)—is associated with cancer and other inflammatory diseases. To date, no enzyme has been documented to exhibit lysophospholipase C activity.

#### 5.2.1. Enzymes with Lysophospholipases A_1_ Activity

Galectin-10: Also known as Charcot-Leyden crystal protein, galectin-10 was first described by Charcot and Robin more than 150 years ago. Galectin-10 is associated with eosinophil- or basophil-mediated inflammation involved with allergy responses [181,182]. Initially, galectin-10 was falsely considered to have weak lipase activity [183] but was later shown to bind a pancreatic-like lysophospholipase in human eosinophils and to inhibit lipolytic activity [184,185]. Highly expressed in eosinophils, galectin-10 is associated with the formation of Charcot-Leyden crystals in lymphocytes; however, the function of the crystals is not fully understood [186].

Phospholipase B-like 1: The membrane-bound protein from neutrophils exhibited weak phospholipase activity for various phospholipids, including LPC [180]; the investigators suggested that phospholipase B-like 1 may play a role in the response against microorganisms and inflammation. Phospholipase B-like 1 is highly expressed on leukocytes in patients with ischemic stroke [187,188], but the detailed mechanisms are not clear.

Lysophospholipase I (encoded by the *LYPLA1* gene) was first cloned from human brain tissue [189,190]. Similar to lysophospholipase I, the paralog lysophospholipase II (encoded by the *LYPLA2* gene) is a cytosolic enzyme that is transported through the cell membrane by palmitoylation [191]. Interference by using small molecules such as palmostatin B inhibits Ras localization and signaling through lysophospholipase acylation [192]. Both lysophospolipase I and II are now classified as EC 3.1.2.22 (UniProt, release 2019_01) and have been renamed acyl-protein thioesterase 1 and 2 (APT-1/APT-2) because they have depalmitoylating activity but low lysophospholipase activity [192,193,194]. Although the alternative names are APT-1/APT-2 and lysophospholipase I/II (LysoPLA I/LysoPLA II), the major functions of these enzymes differ from those of lysophospholipase A_1_ (lysoPLA_1_), which is classified as EC 3.1.1.5. Instead, the depalmitoylating activity of APT-1/APT-2 is associated with membrane protein localization and signaling such as Ras [192].

#### 5.2.2. Enzymes with Lysophospholipases D Activity

Autotaxin: Autotaxin, also called ecto-nucleotide pyrophosphatase/phosphodiesterase-2, is a secreted exo-enzyme that produces most of the extracellular lipid mediator, LPA [195,196]. Autotaxin hydrolyzes phosphodiester bonds of nucleoside triphosphates, lysophospholipids, and cholinephosphate esters [197]. The unique lysophospholipase D activity of autotaxin is determined by a characteristic bimetallic active site and a deep lipid-binding pocket [198]. Originally isolated from human melanoma A2058 cells and defined as an “autocrine motility factor” [199], autotaxin plays a major role in the development of the embryonic vasculature and neural tissue [200,201,202] and in wound healing [203]. However, autotaxin also stimulates tumor cell motility and contributes to the progression of breast cancer [204]. In addition, autotaxin promotes bone cancer metastasis [205], increases the proliferation of thyroid cancer cells [206], and provides cells with resistance to chemotherapy [207]. Moreover, patients with liver fibrosis or hepatocellular carcinoma have increased levels of autotaxin [208,209].

LPA signaling and function: The LPA concentration in plasma ranges between 100 to 164 nM, which is about 1000 times less than that of LPC [210,211,212]. Extracellular LPA is generated via several mechanisms, including the action of phospholipases (group IIA secretory phospholipase A_2_; sPLA_2_-IIA and phosphatidylserine-specific phospholipase A_1_; and PS-PLA_1_) and removal of the choline moiety from LPC by autotaxin [213,214]. Platelets are a major source of LPA because of the presence of autotaxin in their α-granules, and secreted autotaxin is responsible for the basal concentration of LPA in blood [215]. There are six LPA-associated G protein-coupled receptors (GPCRs), LPA_1–6_ or LPAR_1–6_, involved in autotaxin–LPA axis signaling [216,217]. LPA induces a variety of responses such as cell proliferation and migration and cytokine production via GPCR signaling and the effects on ion channels under normal and pathological conditions [218]. LPA plays an important physiological role in the functioning of the immune system. It promotes the homing of lymphocytes to secondary lymphoid tissue through high endothelial venules [219] and stimulates the polarization and transendothelial migration of naïve T cells [220].

LPA and diseases: LPA signaling is associated with metabolic and inflammatory disorders including obesity, insulin resistance, atherosclerosis, and myocardial infarction [221,222,223]. Either by autotaxin overexpression or by supplemented LPA intake, LPA impairs paraoxonase/arylesterase activity and inhibits scavenger receptor BI expression but promotes matrix metalloproteinase-9 activation in THP-1 cells, which results in foam cell formation and atherosclerosis [222,224,225]. Treatment targeting LPA receptors and the downstream signaling pathway attenuates atherosclerosis progression in LDL-receptor deficient mice [226]. In addition, the LPA inactivator, phospholipid phosphatase 3 (PLPP3), is repressed in advanced stages of human atherosclerosis [227]. In mice, inactivation of PLPP3 led to myocardial dysfunction and heart failure [228]. These data indicate that LPA signaling may be an important therapeutic target. LPA stimulates angiogenesis, cancer cell growth, and metastasis [229,230]. High levels of autotaxin/LPA correlate with breast cancer, type I endometrial cancer, and formation of other tumors [231,232,233]. LPA impairs autophagy and regulates tumor progression in various cancer cells [234,235,236,237]. LPA and autotaxin are also highly expressed in the central nervous system, promoting lymphocyte circulation and maturation [219]. Dysregulation of LPA contributes to the pathogenesis of Alzheimer’s disease and mild cognitive impairment in patients with type 2 diabetes [238,239,240].

## 6. Conclusions

LPC, via G protein-coupled receptor signaling, has harmful effects on various cells that include enhancing inflammatory responses, disrupting mitochondrial integrity, and inducing apoptosis. However, the optimal level of LPC in the plasma has not been established, and the mechanisms underlying LPC’s harmful effects are not well understood. Levels of LPC in LDL positively correlate with disease development. An increase in LPC levels is determined primarily by enzyme activity—Lp-PLA_2_ for LPC production. LPCAT contributes to reducing LPC levels; however, the overexpression of LPCAT is associated with cancer. Direct degradation of LPC by autotaxin with lysophospholipase D activity produces LPA, which is another mediator that is highly associated with cancers. Enzymes with lysophospholipaseA1 activity can degrade LPC into harmless materials, but no enzyme with strong lysophospholipase A1 activity has been identified. Targeting LPC may be an important therapeutic option for treating cardiovascular and neurodegenerative diseases. The use of RNAi to inhibit Lp-PLA_2_ or enzyme lysophospholipase A1 activity may potentially be an effective future strategy. In summary, gaining a better understanding of the enzymes and non-enzyme proteins involved in LPC metabolism and how their levels correlate with disease conditions may be useful in identifying novel therapeutic targets for LPC-associated diseases.

## Figures and Tables

**Table 1:** Summary of reported effects of LPC on various cell types.

Cell Type	Effects of LPC	References
Endothelial cells	Induces MCP-1 expression	[17,49]
	Induces inflammatory damage through Notch1 signaling, the overexpression of Notch1, Hes1, and MCP-1	[50]
	Induces MCP-1, IL-8 and RANTES expression through the phosphorylation of ERK1/2, AKT and p38 MAP kinase	[18]
	Induces cytotoxicity/apoptosis and IL-8 production	[19]
	Increases oxidative stress	[21,22]
	Inhibits endothelial cell migration and proliferation	[23,24]
	Impairs endothelium-dependent arterial relaxation	[20]
Adipocytes	Increases IL-1β, IL-6, TNF-α release from adipocytes	[39,40]
Hepatocytes	Disrupts mitochondrial integrity and enhances cytochrome C release	[13]
Immune cells	Induces IFN-γ and TNF-α secretion, immune activation	[16]
	Activates macrophages	[30,34,41]
	Polarizes macrophage activation toward M1 phenotype	[44]
	Activates B cells	[16]
	Induces regulatory T-cell (nTregs) differentiation through Foxp3 expression and TGF-β1 production	[42]
Neuron cells	Impaired the barrier function of the endothelium in the brain microvasculature and induced inflammation	[46]
	Mediates pericyte loss	[47]
	Induces oligodendrocyte demyelination	[45,48]

ERK, extracellular signal-regulated kinase; IFN, interferon; IL, interleukin; LPC, lysophosphatidylcholine; MAP, mitogen-activated protein; MCP-1, monocyte chemoattractant protein-1; TGF, transforming growth factor; TNF, tumor necrosis factor.

**Table 2:** LPC levels in circulation, LDL, or tissue.

Disease	LPC Levels in Plasma or Serum	LPC Levels in LDL Particle	LPC Levels in Tissues
Familial combined hyperlipidemia	1.4× increased [65]	About 1.5× increased LPC concentration in oxidized LDL [65]	N/A
Cardiovascular diseases	LPCs showed an inverse relationship [51,52,53]	About 2× increased LPC concentration in circulating modified LDL [63]	N/A
Diabetes	1.5× increased LPC [4]. Positively associated with blood pressure, carotid artery intima media thickness [70]. Negatively correlated with type 2 diabetes [71,72,73]	1.2–2.8× increased positively correlative with disease progression [49,68]	2-arachidonoyl-lysophosphatidyl-choline increased in atheroma plaques [74]
Myocardial infarction	LPCs 17:0 and LPC 18:2 were selected as biomarkers [62]	N/A	N/A
Stroke	N/A	N/A	LPC 22:6 increased in hippocampus [75]; LPC (16:0) increased in ischemic cerebral regions [76]
Alzheimer’s disease	Plasma level of LPC decreased [54,55]. LPC:PC ratio decreased either in plasma or cerebrospinal fluid [56,77]	N/A	N/A

LDL, low-density lipoprotein; LPC, lysophosphatidylcholine; PC, phosphatidylcholine.

## Abbreviations

AGP	Alpha-1 acid glycoprotein
HDL	High-density lipoprotein
LCAT	Lecithin-cholesterol acyltransferase
LDL	Low-density lipoprotein
LPA	Lysophosphatidic acid
LPC	Lysophosphatidylcholine
LPCAT	Lysophosphatidylcholine acyltransferase
VLDL	Very low density lipoprotein
